# Epistasis in neurotransmitter receptors linked to posttraumatic stress disorder and major depressive disorder comorbidity in traumatized Chinese

**DOI:** 10.3389/fpsyt.2024.1257911

**Published:** 2024-02-29

**Authors:** Ling Xu, Jingyi Zhang, Haibo Yang, Chengqi Cao, Ruojiao Fang, Ping Liu, Shu Luo, Binbin Wang, Kunlin Zhang, Li Wang

**Affiliations:** ^1^ Laboratory for Traumatic Stress Studies and Center for Genetics and BioMedical Informatics Research, CAS Key Laboratory of Mental Health, Institute of Psychology, Chinese Academy of Sciences, Beijing, China; ^2^ Department of Psychology, University of Chinese Academy of Sciences, Beijing, China; ^3^ Academy of Psychology and Behavior, Tianjin Normal University, Tianjin, China; ^4^ People’s Hospital of Deyang City, Deyang, Sichuan, China

**Keywords:** PTSD-MDD comorbidity, gene × gene interaction, single nucleotide polymorphism, neuropeptide Y, dopamine

## Abstract

**Background:**

Posttraumatic stress disorder (PTSD) and major depressive disorder (MDD) comorbidity occurs through exposure to trauma with genetic susceptibility. Neuropeptide-Y (NPY) and dopamine are neurotransmitters associated with anxiety and stress-related psychiatry through receptors. We attempted to explore the genetic association between two neurotransmitter receptor systems and the PTSD–MDD comorbidity.

**Methods:**

Four groups were identified using latent profile analysis (LPA) to examine the patterns of PTSD and MDD comorbidity among survivors exposed to earthquake-related trauma: low symptoms, predominantly depression, predominantly PTSD, and PTSD–MDD comorbidity. *NPY2R* (rs4425326), *NPY5R* (rs11724320), *DRD2* (rs1079597), and *DRD3* (rs6280) were genotyped from 1,140 Chinese participants exposed to earthquake-related trauma. Main, gene–environment interaction (G × E), and gene–gene interaction (G × G) effects for low symptoms, predominantly depression, and predominantly PTSD were tested using a multinomial logistic model with PTSD–MDD comorbidity as a reference.

**Results:**

The results demonstrated that compared to PTSD–MDD comorbidity, epistasis (G × G) *NPY2R*-*DRD2* (rs4425326 × rs1079597) affects low symptoms (*β* = −0.66, *OR* = 0.52 [95% CI: 0.32–0.84], *p* = 0.008, *p_perm_* = 0.008) and predominantly PTSD (*β* = −0.56, *OR* = 0.57 [95% CI: 0.34–0.97], *p* = 0.037, *p_perm_* = 0.039), while *NPY2R*-*DRD3* (rs4425326 × rs6280) impacts low symptoms (*β* = 0.82, *OR* = 2.27 [95% CI: 1.26–4.10], *p* = 0.006, *p_perm_* = 0.005) and predominantly depression (*β* = 1.08, *R* = 2.95 [95% CI: 1.55–5.62], *p* = 0.001, *p_perm_* = 0.001). The two G × G effects are independent.

**Conclusion:**

NPY and dopamine receptor genes are related to the genetic etiology of PTSD–MDD comorbidity, whose specific mechanisms can be studied at multiple levels.

## 1 Introduction

According to a survey conducted across 24 countries by the World Mental Health Organization, approximately 70.4% of adults have experienced at least one traumatic event (1, 2). Subjects witnessing traumatic events have a critical risk of suffering from traumatic stress-related diseases, primarily posttraumatic stress disorder (PTSD) and major depressive disorder (MDD) (3, 4). Previous studies have observed that more than 50% of the adults diagnosed with PTSD have MDD simultaneously (5, 6). Those with PTSD–MDD comorbidity have more treatment burden, lower quality of life, and lower income than the ones with PTSD or MDD alone (6). The mechanism of PTSD–MDD comorbidity has been insufficiently explored. Some scholars believed that PTSD–MDD comorbidity was attributed to the diagnosis symptom overlap between the two (7). Several stable symptoms overlap between PTSD and MDD. These include diminished interest, sleep disturbance, and trouble concentrating in revising all *Diagnostic and Statistical Manual of Mental Disorders* (*DSM*) editions, with unclear evidence. Other scholars believed that PTSD–MDD comorbidity was a different manifestation of a common genetic structure depending on trauma susceptibility. Mundy, Hübel (8) identified that PTSD and MDD could share the genetic structure of trauma susceptibility through polygenic risk scores (PRS) analysis. Nievergelt, Maihofer (9) discovered significant positive correlations between PTSD and MDD genetic variants through PRS. Some researchers observed that the inheritance between PTSD and MDD was entirely related (*r* = 1) (10). A recent meta-analysis indicated that PTSD and MDD share multiple genomic loci through cross-trait (11). Various lines of evidence suggest that genetics may be necessary for PTSD–MDD comorbidity.

PTSD–MDD comorbidity is a complex psychiatric disorder with multiple neural and molecular pathways. Among them, neurotransmitter systems, neuropeptide Y (NPY) receptors, and dopamine play essential roles in mental disorders. Neuropeptide Y consists of 36 amino acids, is widely distributed, and expressed in the central nervous system (CNS), such as the prefrontal cortex, hippocampus, amygdala, etc., and is highly conserved across various species (12–14). The NPY system adapts to stress response (15–17). First, the HPA axis is activated to cope with a stressful situation when people experience a stressful or traumatic event. Afterward, NPY levels would increase in the brain to balance out an overactive stress response (18). The NPY system was dysregulated in PTSD and MDD patients (16), primarily manifested as a decrease. Such downtrend NPY consistently existed at transcription and protein levels in human and animal studies (19, 20). NPY activates biological functions primarily through G protein-coupled receptors, *NPY1R*, *NPY2R*, *NPY4R*, *NPY5R*, and *NPY6R*. Y2R and Y5R are essential in stress response adaptation and emotional occurrence (21–23). Animal and human studies have found that *NPY2R* was upregulated during PTSD and depression (19, 20). Anxiety and depression symptoms in mice were relieved after injecting Y2R antagonists (24), indicating that Y2R may be anxiety-active. Y5R could work with Y1R to inhibit stress-induced dendritic hypertrophy of basolateral amygdala (BLA) pyramidal output neurons, demonstrating anxiolytic and antidepressant effects (25). Therefore, Y2R and Y5R may have opposite roles in stress response adaptation. We conjecture that Y2R and Y5R could be associated with the etiological mechanism of PTSD–MDD comorbidity.

Dopamine is a catecholamine that plays the role of a neurotransmitter in the brain and involves stress adaptation (26). Studies have observed that dopamine was downregulated with reduced activity in MDD and PTSD patients (27, 28). Similar to NPY, dopamine functions through its receptors. There are two main types of receptors, D1-like and D2-like receptors, of which D1-like receptor activation is excitatory (such as D1 and D5 receptors). In contrast, D2-like receptor activation is inhibited (such as D2, D3, and D4 receptors) (29, 30). Chronic exposure to mild stress damages the mesolimbic dopamine circuit, an underlying neurobiological mechanism for anhedonia, a core MDD symptom (28, 31). Established animal studies identified that D2/D3 agonists, not D1 receptors, exert rapid antidepressant effects (32). Early human studies observed that D2/D3 receptor binding was higher in MDD brains than in controls (33). Additionally, D2/D3 agonists could decrease the fear response, indicating significant antidepressant effects in PTSD mice (34). Thus, D2/D3 receptors could be associated with the etiological mechanism behind PTSD. Therefore, we hypothesize that D2/D3 could also be related to PTSD–MDD comorbidity.

There is a complex interplay between NPY and dopamine systems, engaging in the physiological processes of stress adaptation and exerting an impact on the PTSD–MDD comorbidity. The study indicated that NPY neurons in the arcuate nucleus (ARC) extensively projected to the ventral tegmental area (VTA) and accumbens (NAc). They interacted with dopaminergic neural circuits and co-expressed with dopamine, participating in various physiological processes, including stress-induced anxiety (35). Several studies have indicated a positive correlation between NPY activity and dopamine release. NPY and Y5R agonists have been shown to facilitate dopamine release in the NAc and striatum. Y2R antagonists, on the other hand, attenuated NPY-induced dopamine release (36, 37). Furthermore, studies have found an antagonistic interplay between dopamine and NPY in depression and food intake. An increase in dopamine release reduced NPY mRNA and weakened the biological effects induced by NPY (35, 38). Therefore, the dysfunctional antagonistic interplay between NPY and the dopamine system would likely to be involved in the biological mechanisms of related mental disorders. Nevertheless, the precise mechanisms underlying the interaction between the dopamine and NPY systems remain elusive. Similar observations were discerned in relevant genetic studies as well. Genetic polymorphism within the *NPY* have been identified in association with both PTSD and MDD (39, 40). *NPY2R* gene polymorphism, which mediated the effects of NPY, have been found to be associated with stress-induced mental disorders such as MDD (41). The functional variation rs4425326 in *NPY2R* has been reported to be associated with substance abuse (42, 43). Additionally, differential expression of *NPY5R* has been identified in relation to fear learning in PTSD, and lower mRNA levels of *NPY5R* associated with elevated anxiety (44, 45). For the dopamine system, a recent meta-analysis revealed that genetic polymorphism within the *DRD2* exhibited a similar trend in both PTSD and MDD (46). The *DRD3* rs6280 has also been found to be associated with PTSD and MDD (47, 48). In addition to the direct effects of genetic variations, the environment is a crucial risk factor influencing the PTSD–MDD comorbidity. Studies indicated that genetic variations interacted with the environment to impact PTSD and MDD (49, 50). Therefore, it can be speculated that genetic variations in receptor genes, namely, *NPY2R*, *NPY5R*, *DRD2*, and *DRD3*, within the NPY and dopamine systems may directly influence the physiological responses of PTSD–MDD comorbidity or interact indirectly with the environment to impact these responses, but these remain to be explored.

Latent profile analysis (LPA) or latent class analysis (LCA) classifies potential categories for continuous or categorical indicators (51). Traditional factor analysis or disease diagnosis could ignore the differences in disease phenotypes exhibited by heterogeneous groups, decreasing the finding validity. In contrast, LPA considers individual heterogeneity and centers on individuals with potentially identical response patterns that are classified together. This could mitigate the adverse effects of potential individual heterogeneity. Several studies have used LPA to classify individuals with PTSD-related comorbidity. However, the phenotype of PTSD comorbidity with other psychiatric disorders can vary based on the trauma type (52). Contractor et al. (53) used LPA to determine the potential structure of PTSD–MDD comorbidity by analyzing the symptoms of 268 college students with potential trauma events (PTE). PTSD and depression symptoms were divided into three sub-categories: high severity, lower PTSD–higher depression, and higher PTSD–lower depression. The results indicated that PTSD and depression were parallel to each other. Armour et al. (54) conducted LPA on 283 Canadian veterans with PTSD and MDD comorbidity and isolated three similar patterns of comorbidity: high, moderate, and low symptoms of PTSD and MDD. Hruska et al. (55) conducted LPA on 249 motorcycle accident victims with combined PTSD, current MDD, and alcohol or other drug use disorders (MDD/AoDs) and delved into four categories: resilient, mild, moderate, and severe psychopathology. We cannot use these PTSD–MDD patterns directly due to the large sample variation and inconsistent classification results for discerning PTSD and MDD patterns. Therefore, LPA helps determine PTSD and MDD patterns in the current study.

We explored the mechanisms of NPY and dopamine receptors in PTSD–MDD comorbidity in the Chinese population exposed to earthquake-related trauma from a genetic perspective. We analyzed the gene main effects, gene × environment interaction effects (G × E), and gene × gene interaction effects (G × G) to decipher the genetic mechanism in PTSD–MDD comorbidity. The receptor genes *NPY2R*, *NPY5R*, *DRD2*, and *DRD3* were selected as candidate genes. We recruited 1,140 participants from the 2008 Wenchuan earthquake survivors to determine their earthquake-related trauma, PTSD, and depressive symptoms. The participants were divided into four groups through LPA based on the measured PTSD and depressive symptoms. The four categories were low symptoms, predominantly depression, predominantly PTSD, and PTSD–MDD comorbidity.

## 2 Materials and methods

### 2.1 Participants and procedures

All the participants were recruited from people who had survived the 2008 Wenchuan magnitude 8.0 earthquake in Sichuan Province, China. We went to Hanwang District in Mianzhu City, the largest rebuilding community post-earthquake, in November 2013 to obtain the data. Our investigators included trained clinical psychologists, psychiatrists, psychotherapists, and postgraduate psychology students. Sampling procedures were detailed in our previous studies (23). The inclusion criteria were as follows (1): Each family selects one of the most suitable candidates to participate in our study with the family as a research unit (a person in the household born closest to the investigation date). (2) Participants must have experienced the Wenchuan earthquake first-hand. (3) Participants should be at least 16 years of age. (4) Participants should not have suffered from any previous mental disorders. Based on the inclusion criteria, we informed participants of the purpose of our study. We ensured that everyone understood our objectives and obtained their written informed consent. Later, we obtained self-reported questionnaire data from participants, amounting to 1,196 people.

Specialist nurses collected blood samples from these participants for subsequent genotyping. Among them, 24 participants refused to provide blood, 26 failed DNA extraction, and 6 failed genotyping. Finally, we obtained the data for 1,140 participants. The demographic data of participants are demonstrated in **Table 1**. The participants were between 16 and 73 years (*M* = 48.1, *SD* = 10.0), except for two minors (one aged 16 and one aged 17). The rest were adults, with 363 men (31.8%) and 777 women (68.2%), and approximately 99.6% were Han Chinese.

**Table 1:** Demographic information.

Variable	*n* (%)
Gender	
Male	363 (31.8%)
Female	777 (68.2%)
Age	
16–17	2 (0.2%)
Adult	1132 (99.3%)
Missing	6 (0.5%)
Marital status	
Unmarried	148 (13.0%)
Married	990 (86.8%)
Missing	2 (0.2%)
Education level	
Junior high school or below	769 (67.5%)
Senior high school or above	371 (32.5%)
Race	
Han Chinese	1136 (99.6%)
Others	2 (0.2%)
Missing	2 (0.2%)

*N* = 1,140.

This study was conducted by the Declaration of Helsinki and received an ethical committee approval on 12 June 2013 (Institutional Review Board of the Institute of Psychology, Chinese Academy of Sciences, study registered under number H13010). All the participants understood the objectives and signed the written informed consent. The experimental methods were performed following the relevant guidelines.

### 2.2 Measurement

Demographic data were collected using our questionnaire, including the gender, age, marital status, education level, and ethnicity of the participants.

The environmental variable, earthquake-related trauma exposure, was assessed using a questionnaire with 10 questions (23): during the earthquake, (1) Were you trapped under the rubble? (2) Were you injured? (3) Were you disabled due to injuries? (4) Did you participate in rescue efforts? (5) Did you witness the death of someone? (6) Did you see mutilated bodies? (7) Did any family members die during the disaster? (8) Were any family members injured? (9) Did any friend or neighbor die during the disaster? (10) Did you lose your livelihood because of the disaster? Each question was scored at two points; 0 meant no experience, and 1 implied experience. Scores represented the level of earthquake-related trauma exposure. The total score ranged from 0 to 10; the higher the score, the more severe the trauma exposure.

PTSD diagnosis is inferred from the diagnostic criteria provided by the fifth edition of the *Diagnostic and Statistical Manual of Mental Disorders* [*DSM-5*; (56)]. The participant must have experienced at least one traumatic event, one intrusion symptom, one avoidance symptom, two negative alterations in cognition and mood, and two arousal symptoms lasting longer than 1 month. Participant PTSD symptoms were assessed by the *DSM-5* PTSD checklist [PCL-5, (57)]. The questionnaire has 20 self-reported items with four dimensions: intrusion, avoidance, negative alterations in cognition and mood, and hyperarousal. Participants had to answer this questionnaire depending on their circumstances and the Wenchuan earthquake. Each item was scored on a Likert five-point scale from 0 (*not at all*) to 4 (*extremely*). The higher scores represented the severity of the corresponding symptoms. The final score was determined by adding item scores for each dimension. The original PCL-5 questionnaire indicated good reliability and validity (58). The revised Chinese version also possessed good reliability and validity in existing studies (59). Cronbach’s α was 0.95 in this study.

MDD was determined through the Center for Epidemiological Studies–Depression Scale (CES-D) developed by Radloff (60). The questionnaire had 20 self-reported items, evaluating the severity of depressive symptoms from four dimensions: positive affect, depressive affect, somatic complaints, and interpersonal problems. The four questions to evaluate positive affect were reverse score questions. Each item was scored on a Likert four-point scale from 0 (*rare or none of the time/less than one day*) to 3 *(most or all the time/5 to 7 days*). The higher scores were related to more severe depression. The final score was determined by adding item scores for each dimension. The original CES-D questionnaire indicated good psychometric properties (61). The Chinese version of CES-D also had solid reliability and validity (62). Cronbach’s α was 0.87 in our study.

### 2.3 SNP selection and genotyping

Four candidate genes, *NPY2R*, *NPY5R*, *DRD2*, and *DRD3*, and corresponding functionally significant SNPs were selected for our study. For *NPY2R*, rs4425326 was associated with alcohol dependence and withdrawal (43). For *NPY5R*, rs11724320 was related to panic disorder (63). For *DRD2*, rs1079597 has been widely reported to be correlated with addictive behaviors (64, 65). For *DRD3*, rs6280 was associated with impulsive behavior, addiction, and schizophrenia (66, 67).

Based on the DNA extraction standard protocol proposed by Garg (68), DNA was extracted from peripheral blood. The genotyping of rs4425326, rs11724320, rs1079597, and rs6280 was performed using a custom-by-design 2 × 48-Plex SNP scan™ Kit (Genesky Bio-technologies Inc., Shanghai, China) through double ligation and multiplex fluorescence polymerase chain reaction (PCR). Gene Mapper 4.1 (Applied Biosystems) software helped analyze the raw data following the fragment size of allele-specific ligation-PCR products. The genotype call rates exceeded 98%.

### 2.4 Statistical analysis

In our previous study, a detailed LPA for patterns of PTSD and depression symptoms has been reported (59). Specifically, using Mplus 7.0 maximum likelihood estimation with robust standard errors, an LPA was conducted on the *T* scores of eight dimensions, namely, four dimensions of PTSD symptoms (intrusion, avoidance, negative alterations in cognitions and mood, and hyperarousal) and four dimensions of depression symptoms (depressive affect, positive affect, somatic complaints, and interpersonal problems). Comprehensive evaluation of fit indices, parsimony, and interpretability was conducted to select the optimal class model. An LPA model that had a lower Bayesian Information Criterion (BIC), a lower Akaike Information Criterion (AIC), a higher entropy, a significant Lo-Mendell-Rubin likelihood ratio test (LMR LRT), a significant Lo-Mendell-Rubin adjusted likelihood ratio test (ALMR LRT), and a significant bootstrap likelihood ratio test (BLRT) would be the optimal class model. After analyzing, the four-class model exhibited a lower BIC and a lower AIC than the one-, two-, and three-class model, as well as a higher entropy than the five-class model. Simultaneously, the four-class model had significant LMR LRT, ALMR LRT, and BLRT. In accordance with this optimal class model, the collected phenotypic data were synthesized into four groups: low symptoms (*n* = 616, 53.9%), predominantly depression (*n* = 203, 18.2%), predominantly PTSD (*n* = 219, 18.9%), and PTSD–MDD comorbidity (*n* = 102, 9.0%).

We analyzed the variance of each SNP in demographic statistical variables (gender, age, marital status, and education) and earthquake-related trauma exposures using R 4.2.0 (https://www.r-project.org/) to classify the LPA information.

To test whether the main, G × E, and G × G effects among *NPY2R*, *NPY5R*, *DRD2*, and *DRD3* genes were significantly different between PTSD–MDD comorbidity and other groups, multinomial logistic regression was conducted with adjustment for gender, age, marital status, and education level. The independent variables included SNPs, earthquake-related trauma, SNP × SNP, and SNP × earthquake-related trauma. In contrast, the dependent variables were different groups from LPA. In each SNP genotype, the major allele homozygous was coded as 0, heterozygous was coded as 1, and the minor allele homozygous coded as 2. The *p*-values of logistic regression were bidirectional in the analysis. We conducted a permutation test (number of permutations = 100,000) to correct the possible deviation caused by insufficient sample size and multiple comparisons. The results were reported as *p* < 0.05 and permutation *p* < 0.05.

## 3 Results

Based on the descriptive statistical analysis, the mean score of earthquake-related trauma was 3.46 (*SD* = 1.80, score range: 0–10). The mean total score of PTSD was 18.77 (*SD* = 13.46, score range: 1–77), and that of depression was 37.02 (*SD* = 8.63, score range: 20–68), with details in **Table 2**. The analysis of variance revealed that rs4425326, rs11724320, and rs1079597 showed no significant differences in gender, age, marital status, and education. Rs6280 possessed significant differences in age and education level (**Supplementary Table S1**). All the SNP genotypes conformed to the Hardy–Weinberg equilibrium in the all samples, female patients, and male patients, respectively (**Supplementary Tables S2**-**S4**).

**Table 2:** Summary of earthquake-related trauma, depression, and PTSD scores.

	Min	1st quartile	Median	3rd quartile	Max	*M*	*SD*
Earthquake-related trauma	0	2	3	5	10	3.46	1.80
Depression	20	31	36	42	68	37.02	8.63
PTSD	1	9	16	26	77	18.77	13.46

PTSD, posttraumatic stress disorder.

The distribution of SNP genotype in each group is represented in **Table 3**. Multinomial logistic regression analysis of G × G interactions between PTSD–MDD comorbidity and other groups was significant. As described in **Table 4**, *NPY2R*-*DRD2* (rs4425326 × rs1079597) and *NPY2R*-*DRD3* (rs4425326 × rs6280) interaction effects were significant {(*β* = −0.66, *OR* = 0.52 [95% CI: 0.32–0.84], *p* = 0.008, *p_perm_* = 0.008) and (*β* = 0.82, *OR* = 2.27 [95% CI: 1.26–4.10], *p* = 0.006, *p_perm_* = 0.005)} when low symptoms were compared with PTSD–MDD comorbidity. When predominantly PTSD was compared with PTSD–MDD comorbidity, the *NPY2R*-*DRD2* (rs4425326 × rs1079597) interaction effect was significant (*β* = −0.56, *OR* = 0.57 [95% CI: 0.34–0.97], *p* = 0.037, *p*_perm_ = 0.039). The *NPY2R*-*DRD3* (rs4425326 × rs6280) interaction effect was significant (*β* = 1.08, *OR* = 2.95 [95% CI: 1.55–5.62], *p* = 0.001, *p_perm_* = 0.001) when predominantly depression was compared with PTSD–MDD comorbidity.

**Table 3:** Frequencies of different genotypes of each SNP in four groups divided by latent profile analysis.

Group	Low symptom (*n* = 616)	Predominantly depression (*n* = 203)	Predominantly PTSD (*n* = 219)	PTSD–MDD comorbidity (*n* = 102)
rs4425326				
C^a^/C C/T T/T	46 (7.5%) 257 (41.7%) 313 (50.8%)	17 (8.4%) 83 (40.9%) 103 (50.7%)	20 (9.1%) 95 (43.4%) 104 (47.5%)	8 (7.8%) 46 (45.1%) 48 (47.1%)
rs11724320				
C^a^/C C/T T/T	51 (8.3%) 249 (40.4%) 316 (51.3%)	19 (9.4%) 84 (41.4%) 100 (49.3%)	21 (9.6%) 96 (43.8%) 102 (46.6%)	10 (9.8%) 39 (38.2%) 53 (52.0%)
rs1079597				
T^a^/T	103 (16.9%)	36 (17.7%)	44 (20.1%)	23 (22.5%)
T/C	303 (44.2%)	97 (47.8%)	91 (41.6%)	50 (49.0%)
C/C	209 (33.9%)	70 (34.5%)	84 (38.4%)	29 (28.4%)
rs6280				
C^a^/C	54 (8.8%)	20 (9.9%)	20 (9.1%)	5 (4.9%)
C/T	259 (42.0%)	82 (40.4%)	84 (38.4%)	49 (48.0%)
T/T	303 (49.2%)	101 (49.8%)	115 (52.5%)	48 (47.1%)

*N* = 1,140. ^a^ Minor allele. PTSD, posttraumatic stress disorder. MDD, major depressive disorder.

**Table 4:** The G × G effects on PTSD–MDD comorbidity.

	*B*	*SE*	*p*	*p_perm_*	OR (95% CI)
NPY2R × DRD2 (rs4425326 × rs1079597)					
Low symptoms	−0.66	0.25	0.008^**^	0.008^**^	0.52 (0.32, 0.84)
Predominantly depression	−0.50	0.27	0.070	0.071	0.61 (0.36, 1.04)
Predominantly PTSD	−0.56	0.27	0.037^*^	0.039^*^	0.57 (0.34, 0.97)
NPY2R × DRD3 (rs4425326 × rs6280)					
Low symptoms	0.82	0.30	0.006^**^	0.005^**^	2.27 (1.26, 4.10)
Predominantly depression	1.08	0.33	0.001^**^	0.001^**^	2.95 (1.55, 5.62)
Predominantly PTSD	0.35	0.32	0.270	0.277	1.42 (0.76, 2.67)

PTSD–MDD comorbidity was set as reference and compared with low symptoms, predominantly depression, and predominantly PTSD, respectively. The rs4425326 genotype was coded: T/T = 0, C/T = 1, C/C = 2. The rs1079597 genotype was coded: C/C = 0, T/C = 1, T/T = 2. The rs6280 genotype was coded: T/T = 0, C/T = 1, C/C = 2. Gender, age, marital status, and education were covariates. PTSD, post-traumatic stress disorder. MDD, major depressive disorder. SE, standard error. p_perm_, permutation p-value. OR, odds ratio. CI, confidence interval. ^*^p < 0.05, ^**^p < 0.01. Bold indicates significant result.

After putting all the SNPs, G × E, and G × G into one regression model and PTSD–MDD comorbidity was set as the reference, the *NPY2R*-*DRD2* of the rs4425326 × rs1079597 genotype interaction was significant when compared with low symptoms (*β* = −0.68, *OR* = 0.51 [95% CI: 0.31–0.83], *p* = 0.007, *p_perm_* = 0.007) and predominantly PTSD (*β* = −0.59, *OR* = 0.55 [95% CI: 0.33–0.94], *p* = 0.029, *p_perm_* = 0.032). Moreover, the *NPY2R*-*DRD3* of the rs4425326 × rs6280 genotype interaction was significant when compared with low symptoms (*β* = 0.85, *OR* = 2.35 [95% CI: 1.27–4.30], *p* = 0.005, *p_perm_* = 0.005) and predominantly depression (*β* = 0.39, *OR* = 3.09 [95% CI: 1.59–6.03], *p* = 0.0008, *p_perm_* = 0.0009) (**Supplementary Table S5**). Therefore, the interactions of *NPY2R*-*DRD2* and *NPY2R* × *DRD3* were independent.

No SNPs significantly affected PTSD–MDD comorbidity and other groups (*p* > 0.05). Moreover, no significant G × E effects existed between PTSD–MDD comorbidity and other groups (*p* > 0.05) (**Supplementary Table S6**). Additionally, there were no other significant G × G effects (**Supplementary Table S7**).

## 4 Discussion

The current study performed gene main effects, gene–environment interaction, and gene–gene interaction analysis on *NPY2R*, *NPY5R*, *DRD2*, and *DRD3* genotypes in Chinese exposed to earthquake-related trauma. The objective was to explore the potential genetic mechanism of NPY and dopamine receptors in PTSD–MDD comorbidity.

The results did not observe any significant gene main effects and gene–environment interaction. Consistent with previous genome-wide association study (GWAS) results, rs4425326, rs11724320, rs1079597, and rs6280 have not been found to be significantly associated with either PTSD or MDD in GWASs (9, 69). However, this does not necessarily mean that the genotypes of rs4425326, rs11724320, rs1079597, and rs6280 have no effect on PTSD–MDD comorbidity. It is possible that their impact on PTSD–MDD comorbidity is not achieved through a straightforward direct effect. In line with another discovery in the current study, two significant gene–gene interaction effects were identified. With PTSD–MDD comorbidity as a reference group, low symptoms, predominantly depression, and predominantly PTSD group were compared among the four phenotypic groups recommended by LPA. We found *NPY2R × DRD2* and *NPY2R × DRD3* interaction effects significantly, even considering main gene, gene–environment interaction, and gene–gene interaction effects. Therefore, the NPY and dopamine receptor genes do not affect PTSD–MDD comorbidity alone. Instead, they interact with the PTSD–MDD comorbidity mechanism. Consistent with previous findings by Rezitis (35), NPY could interact with the dopamine system in stress response. Specifically, the rs4425326 and rs1079597 allele genotypes interact to put individuals at risk for PTSD–MDD comorbidity compared to low symptoms. In contrast, rs4425326 and rs6280 allele genotypes interact to protect individuals from PTSD–MDD comorbidity. When predominantly PTSD was compared with PTSD–MDD comorbidity, the interaction between rs4425326 and rs1079597 alleles put individuals at risk of developing PTSD–MDD comorbidity. When predominantly depression was compared with PTSD–MDD comorbidity, the interaction between rs4425326 and rs6280 alleles protects individuals from PTSD–MDD comorbidity. These two gene–gene interactions existed after controlling for all gene main effects, gene–environment interaction, gene–gene interaction, earthquake-related trauma exposure, and demographic variables. Our findings established that NPY and dopamine receptors are associated with the genetic etiology of PTSD–MDD comorbidity. Moreover, genetics are involved in the etiology of PTSD–MDD comorbidity (5, 8, 10).

The NPY system is extensively involved in stress response, of which Y2R has been found to be anxiety-active. The rs4425326 polymorphism is in the exon of *NPY2R*, 0.2 Mb upstream on the 5′ end. SNPs in this region may potentially influence the transcriptional regulation of *NPY2R*, thereby affecting the expression of *NPY2R* (70). Studies have shown that Y2R was a presynaptic autoreceptor involved in the NPY negative feedback loop, thereby reducing NPY release to promote anxiety (15).

The dopamine system is widely recognized in psychiatry, manifested by an abnormal decrease of D2/D3 receptors (71). In fact, *DRD2* and *DRD3* shared 52% global homology, suggesting the potential functional similarities in their evolutionary processes, as both belong to the D2-like receptors (72). The rs1079597 is in the regulatory and structural coding regions of *DRD2*. It has been found to be associated with schizophrenia and addictive behaviors (64, 73). The C allele of this SNP was associated with a decreased density of D2 receptors in the individual’s striatum (74). Studies indicated that D2 receptor agonists had anxiolytic and antidepressant effects, alleviating symptoms of PTSD and MDD (34, 75). D2 receptor antagonists have been observed to induce emotional blunting and disrupt the motivational system (76). This phenomenon may be linked to emotional numbness in PTSD and anhedonia in MDD. Therefore, the rs1079597 may alter dopamine release by modulating the density of D2 receptors in the midbrain dopamine circuit, thus participating in the molecular mechanisms underlying the PTSD–MDD comorbidity. The rs6280 is in the exon of *DRD3*, and is a functional SNP that alters dopamine receptor binding affinity (77). Like rs1079597 in *DRD2*, rs6280 was also related to depression and addictive behaviors (48, 78). The rs6280 induced a substitution of serine with glycine at position 9 in the polypeptide product, leading to a substantial increase in the binding affinity of D3 receptors for dopamine (79). This modification facilitated dopamine release in the striatum, thereby contributing to the modulation of the stress adaptation.

The significant interaction between *NPY2R* and *DRD2*, as well as the interaction with *DRD3*, strongly indicates the involvement of the NPY and dopamine systems in PTSD–MDD comorbidity. The rs4425326 is implicated in potentially regulating the expression of *NPY2R*, while rs1079597 modulates D2 receptor density, and rs6280 alters the binding affinity of D3 receptors. Consequently, the interaction between the NPY and dopamine systems in PTSD–MDD comorbidity is likely mediated through the Y2R, D2, and D3 receptors. Y2R and D2 may interact within the amygdala that controls emotional memory, especially fear memory. The memory of traumatic events is a crucial cognitive function influencing PTSD and MDD. Studies have shown that Y2R and D2/D3 receptor activation all facilitated the extinction of fear memory in the amygdala (34, 80). Therefore, Y2R and D2/D3 may exert synergistic effects on the acquisition and extinction of fear memory. The interaction between Y2R and D3 is likely to take place when stress encounters. Studies have shown that when stress had just occurred, Y2R activated the NPY negative feedback loop to decrease NPY release while increasing dopamine release (15, 37). This increased dopamine release may be due to the high affinity of D3 modulated by the rs6280 that prompted VTA dopaminergic neurons to release more dopamine in response to stress (81). Therefore, Y2R and D3 may mobilize the organism in response to stress through synergistic effects. When stress disappeared, the persistent overactivation of Y2R and the compensatory reduction in dopamine within the VTA that failed to disappear may be one of the molecular mechanisms contributing to PTSD–MDD comorbidity. Previous studies have consistently identified a substantial elevation in expression of *NPY2R* mRNA and a decline in dopamine levels in PTSD and MDD (20, 28, 34). Nevertheless, the specific antagonistic or synergistic molecular mechanisms warrant meticulous experimental validation and exploration.

In addition, our study also explored the relationship between *NPY5R* and PTSD–MDD comorbidity. However, there are no significant results. *NPY5R* is not studied enough in PTSD–MDD comorbidity. There were conflicting results in the remaining studies, whose function could not be evaluated during stress adaptation. More research from multiple levels, such as genetics and neurobiology, should explore the future role of NPY and dopamine systems in stress adaptation.

There are several limitations to our study. First, after the LPA, the number of subjects in each group is small, challenging the result validity. In the future, more extensive sample studies should replicate our outcomes. Secondly, the self-reporting method may bring about biases for self-expectation. In the future, techniques such as clinical diagnosis or adding physiological indicators can improve the reliability and validity of the study. Third, the gender ratio in current samples is not balanced, and previous studies have observed an interaction between genetic variants and gender, especially in sexually dimorphic conditions (82). Therefore, further exploration should be conducted in samples with a balanced gender ratio, and the role of gender in PTSD–MDD comorbidity should be further investigated. Finally, our conclusions are limited to the Chinese population exposed to earthquake-related trauma. The same genetic variants may be different in other trauma types or populations. Therefore, the results should be interpreted and generalized with caution.

Overall, the current study identified NPY and dopamine receptor genes involved in the genetic etiology of PTSD–MDD comorbidity. There were significant interactions in two gene pairs, *NPY2R-DRD2* and *NPY2R-DRD3*, providing a new etiological explanation of PTSD–MDD comorbidity. The findings expand the previous research on the anxiolytic and antidepressant effects of NPY and provide information on the dopamine system in stress-related disorders. Site-directed mutagenesis can be further employed to observe the effects of rs4425326, rs1079597, and rs6280 variations on methylation and gene expression in neural cell lines. Moreover, future studies should comprehensively explore the roles of the NPY and dopamine systems in PTSD–MDD comorbidity at multiple levels, taking into consideration the antagonistic, synergistic, or other interactive relationships between NPY and its receptors and dopamine and its receptors. Utilize gene knockout or gene overexpression animal experiments to explore effective genes and their underlying mechanisms. Inject agonists or antagonists to explore specific targets and mechanisms for promoting or alleviating PTSD–MDD comorbidity symptoms. Additionally, further genome-wide interaction analyses on a larger scale are imperative to comprehensively explore and substantiate the contribution of gene interactions to the PTSD–MDD comorbidity at the genome-wide level.

## Data availability statement

The original contributions presented in the study are publicly available. This data can be found here: https://ngdc.cncb.ac.cn/ under the accession number GVM000614.

## Ethics statement

The studies involving humans were approved by Institutional Review Board of the Institute of Psychology, Chinese Academy of Sciences. The studies were conducted in accordance with the local legislation and institutional requirements. Written informed consent for participation in this study was provided by the participants’ legal guardians/next of kin.

## Author contributions

LX: Formal analysis, Investigation, Methodology, Writing – original draft, Writing – review & editing. JZ: Writing – review & editing. HY: Data curation, Writing – review & editing. CC: Data curation, Writing – review & editing. RF: Data curation, Writing – review & editing. PL: Data curation, Writing – review & editing. SL: Data curation, Writing – review & editing. BW: Writing – review & editing. KZ: Conceptualization, Formal analysis, Funding acquisition, Methodology, Project administration, Supervision, Writing – original draft, Writing – review & editing. LW: Conceptualization, Funding acquisition, Investigation, Methodology, Project administration, Supervision, Writing – review & editing.
