# Supplementary material for: Epistasis in neurotransmitter receptors linked to posttraumatic stress disorder and major depressive disorder comorbidity in traumatized Chinese
Source: Front Psychiatry. 2024 Feb 29;15:1257911. doi: 10.3389/fpsyt.2024.1257911 (PMC10937445; doi:10.3389/fpsyt.2024.1257911)
Supplement: Supplementary file 1 [file DataSheet_1.docx]

***Supplementary Material***

**Table S1** Difference analysis of each SNP in control variables.

| **Demography SNP** | **Gender** | **Age** | **Education level** | **Marital status** | **Trauma Total** |
| --- | --- | --- | --- | --- | --- |
| rs4425326 | *F* = 1.67, *P* = 0.20 | *F* = 0.01, *P* = 0.91 | *F* = 0.22, *P* = 0.64 | *F* = 0.63, *P* = 0.43 | *F* = 0.06, *P* = 0.80 |
| rs11724320 | *F* = 0.03, *P* = 0.87 | *F* = 0.11, *P* = 0.74 | *F* = 0.07, *P* = 0.80 | *F* = 0.40, *P* = 0.53 | *F* = 0.01, *P* = 0.91 |
| rs1079597 | *F* = 0.002, *P* = 0.97 | *F* = 0.998, *P* = 0.32 | *F* = 0.07, *P* = 0.79 | *F* = 0.01, *P* = 0.92 | *F* = 0.02, *P* = 0.88 |
| rs6280 | *F* = 0.09, *P* = 0.77 | *F* = 4.04, *P* = **0.04^*^** | *F* = 4.97, *P* = **0.03^*^** | *F* = 0.005, *P* = 0.94 | *F* = 0.39, *P* = 0.53 |

^*^*p* < 0.05. Bold for significant result.

**Table S2** Hardy-Weinberg equilibrium of each SNP in all samples.

| SNP | A1 | A2 | GENO | A1 frequency | observe | expected | $\chi^{2}$ | *p* |
| --- | --- | --- | --- | --- | --- | --- | --- | --- |
| rs1079597 | T | C | 207/541/392 | 0.419 | 0.475 | 0.487 | 0.724 | 0.393 |
| rs11724320 | C | T | 101/468/571 | 0.294 | 0.411 | 0.415 | 0.133 | 0.716 |
| rs4425326 | C | T | 91/481/568 | 0.291 | 0.422 | 0.413 | 0.601 | 0.435 |
| rs6280 | C | T | 99/474/567 | 0.295 | 0.416 | 0.416 | <1×10^-4^ | 1 |

*N* = 1140. A1, minor allele. A2 major allele. GENO, Genotype distribution frequency, A1A1/A1A2/A2A2. Observe, Observation frequency of A1A2. Expected, expected frequency of A1A2.

**Table S3** Hardy-Weinberg equilibrium of each SNP in females.

| SNP | A1 | A2 | GENO | A1 frequency | observe | expected | $\chi^{2}$ | *p* |
| --- | --- | --- | --- | --- | --- | --- | --- | --- |
| rs1079597 | T | C | 142/368/267 | 0.420 | 0.474 | 0.487 | 0.592 | 0.898 |
| rs11724320 | C | T | 75/305/397 | 0.293 | 0.393 | 0.414 | 2.113 | 0.549 |
| rs4425326 | C | T | 61/316/400 | 0.282 | 0.407 | 0.405 | 0.017 | 0.999 |
| rs6280 | C | T | 69/323/385 | 0.297 | 0.416 | 0.417 | 0.011 | 1 |

*N* = 777. A1, minor allele. A2 major allele. GENO, Genotype distribution frequency, A1A1/A1A2/A2A2. Observe, Observation frequency of A1A2. Expected, expected frequency of A1A2.

**Table S4** Hardy-Weinberg equilibrium of each SNP in males.

| SNP | A1 | A2 | GENO | A1 frequency | observe | expected | $\chi^{2}$ | *p* |
| --- | --- | --- | --- | --- | --- | --- | --- | --- |
| rs1079597 | T | C | 65/173/125 | 0.417 | 0.477 | 0.486 | 0.146 | 0.986 |
| rs11724320 | C | T | 26/163/174 | 0.296 | 0.449 | 0.417 | 2.159 | 0.540 |
| rs4425326 | C | T | 30/165/168 | 0.310 | 0.455 | 0.428 | 1.426 | 0.699 |
| rs6280 | C | T | 30/151/182 | 0.291 | 0.416 | 0.412 | 0.028 | 0.999 |

*N* = 363. A1, minor allele. A2 major allele. GENO, Genotype distribution frequency, A1A1/A1A2/A2A2. Observe, Observation frequency of A1A2. Expected, expected frequency of A1A2.

**Table S5** The G × G effects on PTSD-MDD comorbidity (all main, G × E and G × G effects in one model).

|  | ***B*** | ***SE*** | ***p*** | ***p_perm_*** | **OR (95%CI)** |
| --- | --- | --- | --- | --- | --- |
| *NPY2R* × *DRD2*  (rs4425326 × rs1079597) |  |  |  |  |  |
| low symptoms | -0.68 | 0.25 | **0.007^**^** | **0.007^**^** | 0.51 (0.31, 0.83) |
| predominantly depression | -0.48 | 0.28 | 0.088 | 0.093 | 0.62 (0.36, 1.07) |
| predominantly PTSD | -0.59 | 0.27 | **0.029^*^** | **0.032^*^** | 0.55 (0.33, 0.94) |
|  |  |  |  |  |  |
| *NPY2R* × *DRD3*  (rs4425326 × rs6280) |  |  |  |  |  |
| low symptoms | 0.85 | 0.31 | **0.005^**^** | **0.005^**^** | 2.34 (1.27, 4.30) |
| predominantly depression | 1.13 | 0.34 | **0.0008^***^** | **0.0009^***^** | 3.10 (1.59, 6.03) |
| predominantly PTSD | 0.39 | 0.33 | 0.234 | 0.245 | 1.48 (0.77, 2.82) |

PTSD-MDD comorbidity was set as reference and compared with low symptoms, predominantly depression, and predominantly PTSD, respectively. The rs4425326 genotype was coded: T/T = 0, C/T = 1, C/C = 2; rs1079597 genotype was coded: C/C = 0, T/C = 1, T/T = 2; The rs6280 genotype was coded: T/T = 0, C/T = 1, C/C = 2. Gender, age, marital status, and education were as covariates. PTSD, post-traumatic stress disorder. MDD, major depressive disorder. SE, standard error; *p*_perm_, permutation *p* value. OR, odds ratio. CI, confidence interval. ^*^*p* < 0.05, ^**^*p* < 0.01, ^***^*p* < 0.001. Bold for significant result.

**Table S6** Main effects and G × E effects of all SNPs.

|  | ***B*** | ***SE*** | ***p*** | ***p_perm_*** | **OR (95%CI)** |
| --- | --- | --- | --- | --- | --- |
| rs4425326 |  |  |  |  |  |
| low symptoms | -0.15 | 0.18 | 0.387 | 0.389 | 0.86 (0.63, 1.16) |
| predominantly depression | -0.11 | 0.20 | 0.562 | 0.561 | 0.89 (0.71, 1.12) |
| predominantly PTSD | -0.002 | 0.19 | 0.992 | 0.992 | 1.00 (0.99, 1.00) |
| rs4425326 × trauma exposure |  |  |  |  |  |
| low symptoms | 0.10 | 0.10 | 0.303 | 0.305 | 1.10 (0.91, 1.34) |
| predominantly depression | 0.13 | 0.10 | 0.210 | 0.212 | 1.14 (0.88, 1.47) |
| predominantly PTSD | 0.12 | 0.10 | 0.245 | 0.248 | 1.12 (0.89, 1.41) |
|  |  |  |  |  |  |
| rs11724320 |  |  |  |  |  |
| low symptoms | -0.09 | 0.18 | 0.592 | 0.594 | 0.91 (0.76, 1.09) |
| predominantly depression | 0.006 | 0.19 | 0.975 | 0.976 | 1.01 (1.02,1.99) |
| predominantly PTSD | 0.073 | 0.19 | 0.698 | 0.702 | 1.08 (0.93, 1.24) |
| rs11724320 × trauma exposure |  |  |  |  |  |
| low symptoms | 0.06 | 0.10 | 0.526 | 0.548 | 1.06 (0.94, 1.20) |
| predominantly depression | 0.02 | 0.10 | 0.836 | 0.838 | 1.02 (0.98, 1.07) |
| predominantly PTSD | 0.12 | 0.10 | 0.244 | 0.274 | 1.13 (0.89, 1.42) |
|  |  |  |  |  |  |
| rs1079597 |  |  |  |  |  |
| low symptoms | -0.23 | 0.16 | 0.150 | 0.152 | 0.79 (0.51, 1.25) |
| predominantly depression | -0.18 | 0.18 | 0.291 | 0.291 | 0.83 (0.58, 1.19) |
| predominantly PTSD | -0.23 | 0.17 | 0.172 | 0.176 | 0.79 (0.50, 1.25) |
| rs1079597 × trauma exposure |  |  |  |  |  |
| low symptoms | 0.02 | 0.09 | 0.842 | 0.851 | 1.02 (0.98, 1.05 |
| predominantly depression | -0.07 | 0.09 | 0.454 | 0.464 | 0.93 (0.81, 1.07) |
| predominantly PTSD | 0.06 | 0.09 | 0.515 | 0.542 | 1.06 (0.94, 1.19) |
|  |  |  |  |  |  |
| rs6280 |  |  |  |  |  |
| low symptoms | -0.02 | 0.18 | 0.901 | 0.902 | 0.98 (0.94, 1.02) |
| predominantly depression | -0.04 | 0.20 | 0.858 | 0.858 | 0.97 (0.90, 1.03) |
| predominantly PTSD | -0.06 | 0.19 | 0.768 | 0.772 | 0.94 (0.84, 1.06) |
| rs6280 × trauma-exposure |  |  |  |  |  |
| low symptoms | -0.07 | 0.10 | 0.495 | 0.515 | 0.93 (0.82, 1.07) |
| predominantly depression | -0.01 | 0.11 | 0.918 | 0.919 | 0.99 (0.97, 1.01) |
| predominantly PTSD | -0.07 | 0.11 | 0.510 | 0.538 | 0.93 (0.81, 1.07) |

PTSD-MDD comorbidity was set as reference and compared with low symptoms, predominantly depression, and predominantly PTSD, respectively. The rs4425326 genotype was coded: T/T = 0, C/T = 1, C/C = 2; The rs11724320 genotype was coded: T/T = 0, T/C = 1, C/C = 2; rs1079597 genotype was coded: C/C = 0, T/C = 1, T/T = 2; The rs6280 genotype was coded: T/T = 0, C/T = 1, C/C = 2. Gender, age, marital status, and education were as covariates. PTSD, post-traumatic stress disorder. MDD, major depressive disorder. SE, standard error; *p*_perm_, permutation *p* value. OR, odds ratio. CI, confidence interval.

**Table S7** The G × G effects on PTSD-MDD comorbidity (for non-significant results).

|  | ***B*** | ***SE*** | ***p*** | ***p_perm_*** | **OR (95%CI)** |
| --- | --- | --- | --- | --- | --- |
| rs11724320 × rs1079597 |  |  |  |  |  |
| low symptoms | -0.21 | 0.25 | 0.396 | 0.400 | 0.81 (0.54, 1.22) |
| predominantly depression | -0.14 | 0.29 | 0.599 | 0.599 | 0.87 (0.66, 1.15) |
| predominantly PTSD | -0.17 | 0.26 | 0.526 | 0.533 | 0.85 (0.61, 1.17) |
|  |  |  |  |  |  |
| rs11724320 × rs6280 |  |  |  |  |  |
| low symptoms | -0.05 | 0.27 | 0.864 | 0.868 | 0.95 (0.87, 1.05) |
| predominantly depression | -0.27 | 0.30 | 0.369 | 0.371 | 0.76 (0.45, 1.30) |
| predominantly PTSD | 0.046 | 0.29 | 0.876 | 0.876 | 1.05 (0.96, 1.15) |
|  |  |  |  |  |  |
| rs11724320 × rs4425326 |  |  |  |  |  |
| low symptoms | -0.09 | 0.28 | 0.740 | 0.742 | 0.91 (0.76, 1.09) |
| predominantly depression | 0.02 | 0.30 | 0.955 | 0.955 | 1.02 (0.98, 1.05) |
| predominantly PTSD | 0.01 | 0.30 | 0.984 | 0.984 | 1.01 (0.99, 1.02) |
|  |  |  |  |  |  |
| rs1079597 × rs6280 |  |  |  |  |  |
| low symptoms | 0.09 | 0.25 | 0.723 | 0.725 | 1.09 (0.92, 1.30) |
| predominantly depression | -0.27 | 0.28 | 0.342 | 0.346 | 0.77 (0.46, 1.29) |
| predominantly PTSD | -0.15 | 0.27 | 0.588 | 0.590 | 0.86 (0.64, 1.15) |

PTSD-MDD comorbidity was set as reference and compared with low symptoms, predominantly depression, and predominantly PTSD, respectively. The rs4425326 genotype was coded: T/T = 0, C/T = 1, C/C = 2; The rs11724320 genotype was coded: T/T = 0, T/C = 1, C/C = 2; rs1079597 genotype was coded: C/C = 0, T/C = 1, T/T = 2; The rs6280 genotype was coded: T/T = 0, C/T = 1, C/C = 2. Gender, age, marital status, and education were as covariates. PTSD, post-traumatic stress disorder. MDD, major depressive disorder. SE, standard error; *p*_perm_, permutation *p* value. OR, odds ratio. CI, confidence interval.
